# Facial emotion mimicry in older adults with and without cognitive impairments due to Alzheimer's disease

**DOI:** 10.3934/Neuroscience.2021012

**Published:** 2021-01-27

**Authors:** Justyna Gerłowska, Krzysztof Dmitruk, Konrad Rejdak

**Affiliations:** 1 Department of Educational Psychology and Psychological Assessment, Institute of Psychology University of Maria Skłodowska-Curie, Lublin, Poland; 2 Institute of IT, University of Maria Skłodowska-Curie, Lublin, Poland; 3 Department of Neurology, Medical University of Lublin, Lublin, Poland

**Keywords:** emotion, aging, cognitive impairment, Alzheimer's disease, mild cognitive impairments

## Abstract

Facial expression of humans is one of the main channels of everyday communication. The reported research work investigated communication regarding the pattern of emotional expression of healthy older adults and with mild cognitive impairments (MCI) or Alzheimer's disease (AD). It focuses on mimicking of displayed emotional facial expression on a sample of 25 older adults (healthy, MCI and AD patients). The adequacy of the patients' individual facial expressions in six basic emotions was measured with the Kinect 3D recording of the participants' facial expressions and compared to their own typical emotional facial expressions. The reactions were triggered by mimicking 49 still pictures of emotional facial expressions. No statistically significant differences in terms of frequency nor adequacy of emotional facial expression were reported in healthy and MCI groups. Unique patterns of emotional expressions have been observed in the AD group. Further investigating the pattern of older adults' facial expression may decrease the misunderstandings and increase the quality of life of the patients.

## 1. Introduction

The facial expression of emotional information is perceived as natural way of communicating the inner states of the human being. It also enriches the conversation without the additional verbal cues. It is generally perceived as a natural and automatic function but in certain clinical conditions it is observed to change [1]–[4]. One of these cases is Alzheimer's disease (AD) [5] and its prodromal phase mild cognitive impairments (MCI) [6]–[8]. Even though that general criteria of the both conditions highlight the cognitive aspect of disturbances [9] clinicians and caregivers report substantial impact of the altered facial emotional expression to the everyday caregiver-patient contact. The presence of the emotion dysregulation in everyday contact significantly impacts the welfare of the informal caregivers and is connected with decreased quality of life of the patient's family system [10]–[13]. Based on the authors' clinical experience the herein study has been performed. The main goal was the initial observation to see if emotional facial mimicry within the older adults had been altered due to the coexistence of the increasing cognitive impairments due to dementia. The authors focused on exploring the potential relation between the cognitive functioning level, verbal fluency, emotion perception, and the adequacy of the facial mimicking of the emotional stimuli. The study was approved by the Medical University of Lublin ethical committee on human research.

## 2. Materials and methods

### 2.1. Inclusion and exclusion criteria

For the purpose of the study 65 older adult volunteers (45 women and 20 men) were invited to participate in the study. They had normal or corrected to normal vision and hearing. The main inclusion criteria were: age (70–90 years old); signed informed consent for the participation in the study; maintained language skills; fulfilling the criteria of the cognitively healthy older adult; MCI and AD patient according to [9]; and confirmed medical diagnosis of AD (only in case of AD group). The main excluding criteria were: observed currently or in the past coexistence of the neurological condition (such as: epilepsy, tumor, stroke or brain damage due to trauma); and psychiatric conditions (such as depression) altering the cognitive state.

### 2.2. Neuropsychological assessment

A twofold neuropsychological assessment was performed to verify the cognitive, emotional and general health status. The first meeting was devoted to screening procedures (Mini Mental State Examination, Clinical Dementia Rating scale, Global Deterioration Scale, 7-minute test, fluency tasks, Hachinsky's scale, and the semi-structured interview). Participants fulfilling the inclusion/exclusion criteria then underwent the second part of the assessment consisting of Right Hemisphere Lesion Battery (RHLB-PL). Subjects showing cognitive impairment lower than 10 MMSE points were rejected from the further examination. Subject reporting more than 5 points in Hachinsky's scale were excluded from the study. Subjects not fulfilling the inclusion/exclusion criteria were briefed on their current condition and thanked for the participation.

### 2.3. Statistical analysis

Subjects fulfilling the criteria were invited to participate in the experiment on emotional facial expressions. The final group of participants in the experiment consisted of 25 right-handed older adults [M = 81.75 yrs. (73.58–89.66), SD = 4.17]. The group consisted of healthy older adults (6 women and 3 men), MCI patients (6 women and one men) and AD patients (6 women and 3 men). The groups detailed description is presented in Table 1 and Table 2. The normality of results' distribution was verified with W Shapiro-Wilk. Normality of distribution was confirmed for: age; MMSE; fluency (K, animals and body parts); RHLB- humor; and RHLB- emotional prosody. The homogeneity of results was verified with the Levene test. Depending on the results of the above the further analysis was performed with ANOVA, Kruskal-Wallis; or U-Mann-Whitney, and Chi^2^.

**Table 1.** Description of the sample.

	Group	M	SD	
Age (years)	Healthy	82.5	4.89	F_(2, 22)_ = 0.39; p = 0.68
	MCI	80.62	4.43	
	AD	81.88	3.42	
Education (years)	Healthy	12.67	3.16	Chi^2^ = 1.65; p = 0.44
	MCI	10.57	3.41	
	AD	11.56	2.96	
Hachinsky's scale (points)	Healthy	1.9	0.9	Chi^2^ = 8.92; p = 0.01
	MCI	2.7	1.1	
	AD	3.6	1.0	
Time from the diagnosis (years)	MCI	1.29	0.76	U = 10.5; p = 0.02
	AD	3.11	1.9	

Note: MCI: mild cognitive impairment; AD: Alzheimer Disease; M: mean; SD: standard deviation.

The groups did not show differences in terms of age and education level; but differed in terms of Hachinsky's scale. The post hoc analysis showed the significant differences between healthy and AD group (U = 8.5; p = 0.004) but not between healthy and MCI (U = 17.5; p = 0.18), nor AD and MCI group (U = 19; p = 0.17). The clinical groups differed in terms of the time from the initial diagnosis.

The groups were well selected, which was confirmed by the significant differences in the main neuropsychological scales (MMSE, 7-minutes test). The groups of subjects showed significant differences in all neuropsychological scales. The differences correspond with meeting the criteria of healthy, MCI and AD group. The post hoc analysis showed the significant differences between healthy and AD patients in all scales used. The differences between healthy controls and AD patients in fluency tasks are: letter K (p = 0.006), animals (p < 0.001), body parts (p < 0.001), and letter F (U = 8; p = 0.004). The differences between the healthy participants and AD patients in RHLB subscales are: humor (U = 19; p = 0.05), lexical prosody (U = 7.5; p = 0.003), and emotional prosody (p = 0.001).

The significant differences between the healthy and MCI patients were observed in: fluency animals (p = 0.004), and body parts (p = 0.03). The differences between the healthy and MCI patients at the level of tendency were observed in: fluency letter F; RHLB humor; and RHLB lexical prosody. The significant differences between MCI and AD patients were observed in RHLB emotional prosody (p = 0.04) only. The differences between MCI and AD patients at the level of tendency were observed in: fluency body parts; and RHLB lexical prosody.

**Table 2.** Neuropsychological profile of the participants.

Scale	Group	M	SD	
MMSE	Healthy	30.7	2.31	F = 77.65; p = 0.001
	MCI	23.1	3.4	
	AD	15.8	1.9	
Fluency-K	Healthy	10.4	2.8	F = 6.12; p = 0.008
	MCI	7.7	4.2	
	AD	4.4	3.9	
Fluency-F	Healthy	8.2	2.8	Chi^2^ = 9.33; p = 0.009
	MCI	4.7	4.2	
	AD	2.3	3.1	
Fluency-animals	Healthy	15.5	4.4	F = 17.22; p = 0.0001
	MCI	8.6	3.6	
	AD	5.2	3.2	
Fluency-body	Healthy	19.8	5.2	F = 15.43; p = 0.0001
	MCI	13.1	3.9	
	AD	7.4	4.8	
7-minutes test	Healthy	0.1	0.33	Chi^2^ = 19.82; p = 0.0001
	MCI	2.57	1.13	
	AD	3	0	
RHLB-PL				
Humor	Healthy	6.2	2.4	Chi^2^ = 5.08; p = 0.08
	MCI	3.9	1.7	
	AD	3.9	0.8	
Lexical prosody	Healthy	12.3	3.7	Chi^2^ = 10.13; p = 0.006
	MCI	9.1	2.9	
	AD	5.6	4.1	
Emotional prosody	Healthy	10	2.9	F = 9.97; p = 0.001
	MCI	8.1	2.5	
	AD	4.7	2.2	

Note: MCI: mild cognitive impairment; AD: Alzheimer Disease; M: mean; SD: standard deviation; MMSE: Mini Mental State Examination; RHLB-PL: Right Hemisphere Lesion Battery-Polish version.

### 2.4. Facial emotions mimicking experiment layout

The subjects were presented with 83 pictures of human faces (male and female) cropped to show only the face of the actor. The actors were young and old Caucasian adults of both sexes without any distinguishing facial features (e.g. glasses, mustaches or beards). The pictures were taken from P. Ekman's Pictures of facial affect battery (POFA) together with pictures specially prepared by the authors. Pictures were assessed by 11 psychologists on the matter of emotional content. The psychologists were required to name the emotion presented and give its intensity in percentage value. The Fleiss' Kappa factor was calculated for each picture from the following categories: happy, sad, fear, disgust, surprised and anger. Pictures with the factor higher than 0.6 were included in the further analysis. In total the data obtained for the display of 15 color and 34 black-white pictures was analyzed. The data collected for the rest of the pictures was discarded from the further analysis. The subjects were blind to the researchers' choice and were instructed to react to all 83 pictures “as well as they can”. The subjects' task was to “Do as they do (presented actors)”. The majority of participants spontaneously gave the name of the emotion presented. Their assessment was mostly adequate. Each part of the assessment took approximately 45 minutes.

**Figure 1.**
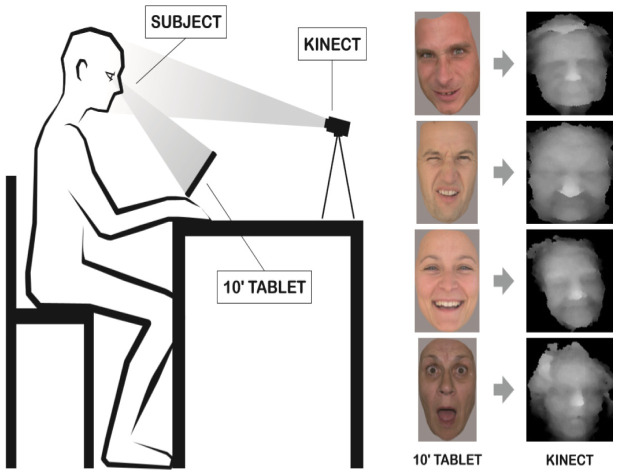
Study layout with the example of stimuli (angry/disgust/happy/surprise) and their corresponding 3D recording.

The subjects were sitting in a quiet room and were not disturbed throughout the experiment. The researcher was present through the whole experiment and if necessary, focused the participants' attention on the stimuli. Subjects were instructed to look at the tablet where the pictures were presented. Behind the tablet the Kinect sensor was placed at 1.5 m distance. The experiment setting is shown in Figure 1. The period of picture presentation was automatically set for one minute with the interval of 15 seconds, where the black background was displayed between the stimuli. The presentation of the stimuli was signaled by the cross in the middle of the screen. The average interval of data collection for healthy participants was set for the 10–15 seconds of initial picture display which was sufficient to react to single stimuli. In case of MCI/AD participants the interval for data collection was 10–15 seconds from the moment of stimuli recollection. It was possible to manually shorten the time of the stimuli presentation if the participant reacted to the stimuli presented. If no reaction was recorded within 1 minute the next stimuli was presented. Many participants during the 15 seconds interval between stimulus presentation used the tablet as a mirror checking on their appearance (fixing the hair, or commenting on their appearance). Only two participants felt discomfort during the emotional stimulus presentation and resigned from further participation. All other participants reported the experiment as amusing and not intrusive.

The data was collected through out all the experiment which on average took 45 minutes. At the beginning the subjects were asked to recall the happy, sad, annoying and pleasant surprises in their life in order to establish the individual personalized profile of the emotional facial expression of each participant in six basic emotions. The neutral facial expression was established too.

The face of the subject was continuously mapped by the Kinect sensor. The subjects' typical emotional facial reactions representing six basic emotions and neutral expression were set as the individual subject's prototypical facial expression. The facial emotional mimicry was measured by the specially designed algorithm comparing the subject's facial reaction registered within 10–15 seconds of time locked recording, corresponding to the stimulus and compared to the prototypical subject's individual reaction in corresponding to emotional category. The exact algorithm is described in [14].

### 2.5. Ethics approval of research

The research was conducted ethically in accordance with the World Medical Association Declaration of Helsinki. The subjects (or their guardians) have given their written informed consent and that the study protocol was approved by the Medical University of Lublin ethical committee on human research.

## 3. Results

**Figure 2.**
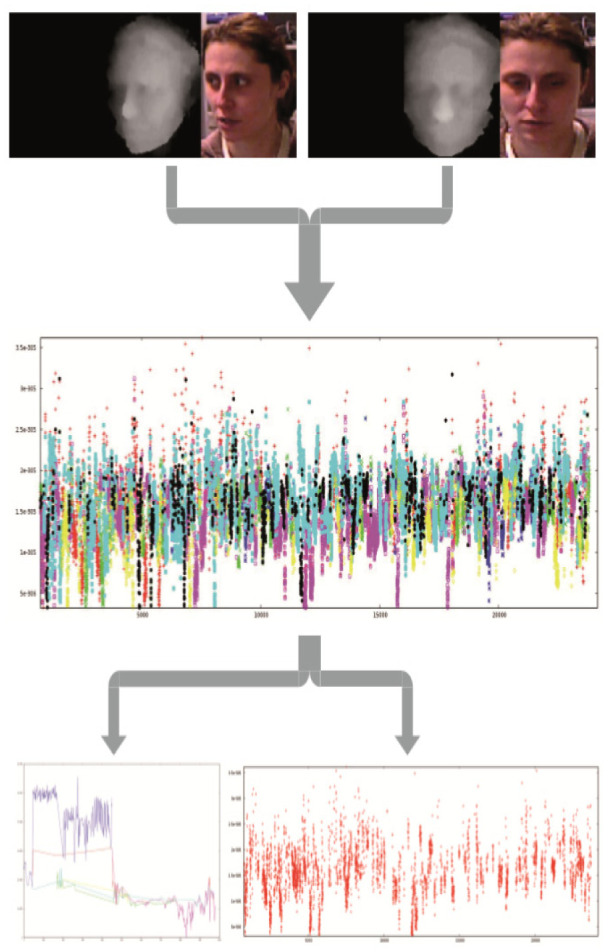
Emotion mimicking data processing: 1. Data recording in threefold frames (stimuli/3D screenshot/RGB screenshot); 2. The facial emotions accuracy during the experiment (red-neutral, green-fear, navy blue-anger, pink-sad, yellow-disgust, blue-happy, black-surprise) 3. Facial emotional reaction to one stimuli (left) and the distribution of the happy facial emotional reaction during the whole experiment (right).

The final analysis was performed for 21 participants (seven subjects in each group: healthy older adults, MCI patients, AD patients). The data of 4 participants was discarded due to high level of noise (N = 2) or withdrawal of consent for the experiment (N = 2). During the recording 6000–74000 pictures were collected for each participants. Only the event locked data collected in the 10–15 seconds of the most intense reaction to the selected 49 pictures was analyzed. The initial automatic assessment was performed, but due to differences in reaction times of healthy older adults and MCI/AD patients the manual selection of the peak emotional responses was used for the further analysis. The graphical presentation of the emotional adequacy of participant's reaction is shown in Figure 2. The selected frames of the emotional facial reactions performed by the participant were assessed in terms of similarity to prototypical facial expression.

The participants' emotional reactions to all 49 stimuli were calculated in terms of finding the differences between the groups favoring the particular emotion. The groups did not show statistically relevant differences in frequency of emotions expressed. The participants' emotional facial expression adequacy to the stimuli main emotion was similar for all the groups. The reactions were later clustered based on the sign of the emotion to positive and negative stimuli. The neutral stimuli were not included. The detail information is presented in Table 3. The adequacy of the emotional facial mimicking of the stimuli was calculated in the mixed model of variance. No statistical differences were observed between the groups (F_(1;18)_ = 0.02; p = 0.98), nor main effects were identified (F_(1;18)_ = 1.85; p = 0.19), nor their interactions (F_(2;18)_ = 0.44; p = 0.65). Mauchly's sphericity test wasn't confirmed. Homogeneity of the variance was confirmed with M Box test. The further analysis for the dependent samples was conducted to verify the differences between the adequacy of the positive and negative stimuli emotional facial expression within the groups. The normality of distribution was confirmed with W Shapiro-Wilk. The statistically significant negative correlation (r-Pearson) between adequacy of the emotional facial expression in response to emotional stimuli has been confirmed (r = −0.41; p = 0.06).

**Table 3.** Facial emotional mimicking adequacy among the groups.

Emotion	Mean frequency of emotional reaction	SD		% of adequacy of emotional reaction	SD	
Happy healthy	11	7.1	F = 0.27; p = 0.77	20.9	11.5	F = 1.53; p = 0.24
MCI	6.43	4.9		16.5	16.3	
AD	11.57	5.9		20.9	10.6	
Fear healthy	8.86	3.8	F = 0.5; p = 0.62	10.7	13.4	F = 0.47; p = 0.63
MCI	6.86	5		17.9	18.9	
AD	6.29	6.5		10.7	13.4	
Disgust healthy	9	4.4	F = 0.29; p = 0.75	17.9	17.5	F = 0.084; p = 0.36
MCI	5.42	4.7		23.2	18.3	
AD	9.14	6.7		16.1	18.7	
Anger healthy	6.43	3.7	F = 1.23; p = 0.32	12.5	14.4	F = 0.16; p = 0.86
MCI	5.86	4.1		12.5	14.4	
AD	5	6.3		3.6	6.1	
Surprise healthy	4	3.2	F = 0.91; p = 0.42	5.4	9.8	Chi^2^ = 4.91; p = 0.086
MCI	10	10.2		19.6	25.9	
AD	10	6		16.1	22.5	
Sad healthy	6.71	4.6	F = 0.22; p = 0.81	10.7	15.2	F = 0.16; p = 0.86
MCI	7.71	5.9		12.5	14.4	
AD	8.57	7.8		19.6	25.9	
Neutral healthy	10.57	4.1	F = 2; p = 0.16			
MCI	13.86	9.1				
AD	6.71	5.9				
Emotions Total healthy				14	3.8	
MCI				16.9	4.8	
AD				15.5	4.1	
						
Negative emotions healthy				13.27	6.1	
MCI				16.33	11.3	
AD				12.76	5.8	
						
Positive emotions healthy				20.88	11.5	
MCI				16.48	16.3	
AD				20.88	10.6	

Note: MCI: mild cognitive impairment; AD: Alzheimer Disease; SD: standard deviation.

The post hoc analysis revealed the significant correlation for AD patients (r = −0.81; p = 0.02). No statistically significant correlations were confirmed within the remaining groups. The presented results suggest that despite the same frequency of negative/positive emotions mimicked in all groups the AD group shows significant difficulties with flexible adjustment to the sign of the stimuli presented. The results show that in AD group participants were able to adequate represent only one sign of emotional stimuli, either positive or negative.

## 4. Discussion

The obtained results partially correspond with the current data on the abilities of emotion recognition within the groups of older adults [7],[15]–[18]. The level of observed adequacy of the emotional facial expression may be connected with the level of the emotion expression intensity presented. Such a result has been already reported within the MCI and AD patients [5]. The natural mimicking and emotional contagion of the emotions presented is less efficient in persons with cognitive disorders [1]–[8]. As well it has been reported that neutral facial expressions of older adults may be identified as more negative than the actor of conversation intended [19]. The facial emotional mimicking is an unconscious social glue connecting us with our conversational partners [20]. The underlying mechanisms are complex and are being gradually discovered [21]–[29]. It is also believed that the network responsible for correct perception and performance of facial emotional reactions is fragile and may be changed during aversive individual experiences [30]–[34]. Participants of the current study were children during the II World War which may have leave its mark on their abilities to read facial expressions. As well the noticeable right hemisphere deficits in AD group may correspond with the lower abilities to express and recall the emotional content. As well the bias in responding to the positive and negative emotional stimuli has been observed within heathy young adults [35] and increases with age [14]. The above may have influenced the results of the current study and could be the typical pattern of emotional functioning of the older persons with and without cognitive dysfunctions.

The reported herein results should be treated as an initial findings. The further analysis with the bigger samples could reveal more significant and complex results within all the groups: healthy, MCI and AD.

The technological possibility of recording and analyzing dynamic changes in emotional human expression has its limitations [36]–[39]. Further developments within this field are expected soon. The current lines of investigation cover: physiological reactions measured by multiple sensors (Empatica E4) [40]–[41]; behavioral reactions measured by vison sensors (Kinect) [42]; and mapping the brain networks with deep learning systems [43]–[45]. The fast progress in the interdisciplinary research introducing the fusion of novel usage of already discovered solutions may give more light on human emotional functioning [39]. Presented herein the protocol of the study gives initial glimpse on the future usage of vision sensors in clinical setting. It is probable that observed level of adequacy in emotion mimicking may be connected with the sensitivity of the sensors applied in the study.

All of the discussed studies used language in establishing the sign and level of the emotion reported by the subjects. The self-reported questionnaires and labeling the emotions have been applied. The AD and MCI progression typically is connected with the loss of verbal fluency and the adequate use of mother tongue. Therefore, the results of the cited studies can be obtained only in the groups with relatively preserved language skills. The current study is one of few to the authors' knowledge that applied the nonverbal way of communication the emotion recognition in other humans' faces. Due to its resemblance to everyday human interactions, it was perceived by the subjects as pleasant and non-threatening. The reported results show similar pattern as already described in recent studies but were possible to obtain without specialized equipment (fMRI, EEG) and without the use of language. With the technological changes it may become possible to observe the dynamic changes of the alternation in the human facial expression caused by the disease. With the increase of application using the facial recognition embedded in the smartphones self-diagnosis of possible cognitive or mood disorder would become something more than just a vision of the future.

## 5. Conclusions

Observed changes in the demographic structure of societies worldwide and the growing amount of old persons suffering from the dementia and mild cognitive impairment bring up the question of self-diagnosing. The typical neuropsychological assessment performed by the professionals covers one edge of the everyday functioning. The emotional functioning assessment is currently based on observation and questionnaires. The data collected during such assessment is partial and subjective therefore developing the more objective tool that would enable measuring the process of emotional mimicking is needed. The recent development of the mobile technology and the vision capture algorithms give the hope for the caregivers to develop the application for the emotional mimicking adequacy recognition. Such application could give the start for the future development of the rehabilitation of the facial emotional recognition.
